# Effect of nitrogen fertilizer on the resistance of rice near-isogenic lines with BPH resistance genes

**DOI:** 10.1186/s40529-022-00347-8

**Published:** 2022-05-23

**Authors:** Shau-Ching Lin, Yi Li, Fang-Yu Hu, Chih-Lu Wang, Yun-Hung Kuang, Chang-Lin Sung, Shin-Fu Tsai, Zhi-Wei Yang, Charng-Pei Li, Shou-Horng Huang, Chung-Ta Liao, Sherry Lou Hechanova, Kshirod K. Jena, Wen-Po Chuang

**Affiliations:** 1 Department of Agronomy, National Taiwan University, Taipei, 10617 Taiwan; 2 Crop Improvement Division, Taoyuan District Agricultural Research and Extension Station, Council of Agriculture, 32745 Taoyuan City, Taiwan; 3 Crop Science Division, Taiwan Agricultural Research Institute, Council of Agriculture, Taichung City, 413008 Taiwan; 4 Department of Plant Protection, Chiayi Agricultural Experiment Station, Taiwan Agricultural Research Institute, Council of Agriculture, Chiayi, 60044 Taiwan; 5 Crop Environment Division, Taichung District Agricultural Research and Extension Station, Council of Agriculture, Changhua County, 51544 Taiwan; 6 Novel Gene Resources Laboratory, Strategic Innovation Platform, International Rice Research Institute, DAPO Box 7777, Metro Manila, Philippines; 7 School of Biotechnology, Kalinga Institute of Industrial Technology, Bhubaneswar, 751024 Odisha India

**Keywords:** *Nilaparvata lugens*, Nitrogen, *BPH9*, *BPH17*, *BPH32*

## Abstract

**Background:**

Nitrogen is an essential macronutrient for plant growth and development. Crops with a high nitrogen input usually have high yields. However, outbreaks of brown planthoppers (*Nilaparvata lugens*; BPH) frequently occur on rice farms with excessive nitrogen inputs. Rice plants carrying BPH resistance genes are used for integrated pest management. Thus, the impact of nitrogen on the resistance of rice near-isogenic lines (NILs) with BPH resistance genes was investigated.

**Results:**

We tested these NILs using a standard seedbox screening test and a modified bulk seedling test under different nitrogen treatments. The amount of nitrogen applied had an impact on the resistance of some lines with BPH resistance genes. In addition, three NILs (NIL-*BPH9*, NIL-*BPH17*, and NIL-*BPH32*) were further examined for antibiosis and antixenosis under varying nitrogen regimes. The *N. lugens* nymph population growth rate, honeydew excretion, female fecundity, and nymph survival rate on the three NILs were not affected by different nitrogen treatments except the nymph survival rate on NIL-*BPH9* and the nymph population growth rate on NIL-*BPH17*. Furthermore, in the settlement preference test, the preference of *N. lugens* nymphs for IR24 over NIL-*BPH9* or NIL-*BPH17* increased under the high-nitrogen regime, whereas the preference of *N. lugens* nymphs for IR24 over NIL-*BPH32* was not affected by the nitrogen treatments.

**Conclusions:**

Our results indicated that the resistance of three tested NILs did not respond to different nitrogen regimes and that NIL-*BPH17* exerted the most substantial inhibitory effect on *N. lugens* growth and development.

## Background

Host plant resistance is a valuable resource for integrated pest management (IPM). Plants with resistance reduce not only herbivore damage but also pesticide usage. Antibiosis, antixenosis, and tolerance are the three categories of host plant resistance (Painter 1951; Smith 2005). Plants with antibiosis traits affect insect survival, whereas plants with antixenosis may influence insect behavior (Smith 2005). Plant tolerance is a unique trait in which a plant can withstand herbivore damage but does not affect insect growth and behavior (Smith 2005). Currently, insect-resistant varieties of major crops (rice, wheat, etc.) are widely used in IPM programs (Cohen et al. 1997; Jlibene and Nsarellah 2011; Nsarellah et al. 2003; Peñalver Cruz et al. 2011).

The brown planthopper (BPH), *Nilaparvata lugens* (Stål), is the major rice pest threatening rice production. *N. lugens* causes plant mortality symptom “hopper burn” and transmits plant viruses, such as grassy and ragged stunt viruses. Thirty nine BPH resistance genes in rice have been identified (Zhang et al. 2020). Twenty of them have been found in rice cultivars, whereas some have been identified in wild rice species, such as *Oryza australiensis*, *O. officinalis*, *O. minuta*, *O. rufipogon*, *O. glaberrima*, and *O. nivara* (Du et al. 2020). Furthermore, 14 BPH genes located on chromosomes 3, 4, 6, and 12 have been cloned and characterized (Cheng et al. 2013; Du et al. 2009, 2020; Guo et al. 2018; Ji et al. 2016; Liu et al. 2015; Ren et al. 2016; Tamura et al. 2014; Wang et al. 2015; Zhao et al. 2016). For example, *BPH9* was found in the rice variety Pokkali and encodes a coiled-coil, nucleotide-binding, nucleotide-binding, and leucine-rich repeat domain (CC-NB-NB-LRR) protein (Zhao et al. 2016). *BPH17* has been found in the rice cultivar Rathu Heenati and identified as a cluster of lectin receptor kinases (Liu et al. 2015). *BPH32* was identified in PTB33 and contains a short consensus repeat (SCR) domain (Ren et al. 2016).

In our previous study, twelve near-isogenic lines (NILs) carrying one or two BPH resistance gene(s) were evaluated for resistance to environmental changes (high air temperature and high carbon dioxide concentration) (Kuang et al. 2021). Two of nine NILs with a single BPH resistance gene (*BPH17* and *BPH20*) and two of three NILs pyramided with two BPH resistance genes (*BPH9 + 32* and *BPH18 + 32*) maintained resistance against *N. lugens* under environmental changes (Kuang et al. 2021). Furthermore, NIL-*BPH17* exerted a strong inhibitory effect on *N. lugens* growth and development despite the environmental changes. In addition, plants with the *BPH17* resistance gene show resistance against the white-back planthopper [*Sogatella furcifera* (Horváth)] (Liu et al. 2015). BPH resistance genes are currently used in breeding programs for insect-resistant rice (Du et al. 2020; Jena et al. 2017; Nguyen et al. 2019; Xiao et al. 2016).

Since insect herbivores mainly obtain nutrients from host plants, the resource availability of the host plant is the main factor affecting insect herbivore growth and development (Awmack and Leather 2002). Nitrogen is an essential macronutrient for plant growth and development. Generally, crops with high nitrogen input have high production. However, nitrogen is also the limiting nutrient for insect herbivores. Insect herbivores feeding N-enriched host plants show enhanced fitness (Lu and Heong 2009; Lu et al. 2004; Prestidge 1982; Wier and Boethel 1995). For example, rice water weevils (*Lissorhoptrus oryzophilus*) feeding on high-nitrogen-treated plants showed increased adult feeding and oviposition preferences (Jiang and Cheng 2003). Furthermore, to obtain sufficient nitrogen, the midgut of Lepidoptera can digest large amounts of plant proteins, including Rubisco (Bhardwaj et al. 2014).

*N. lugens* outbreaks frequently occur on rice farms with excessive nitrogen input (Visarto et al. 2001). By increasing the host plant’s nitrogen content, insects may obtain sufficient nutrients to overcome plant resistance. Thus, we aimed to determine whether *N. lugens* feeding on rice plants with BPH resistance genes under high nitrogen input would overcome resistance. Therefore, we used twelve NILs with BPH resistance genes developed by the International Rice Research Institute (IRRI) to evaluate the impacts of nitrogen on resistance (Jena et al. 2017). These NILs were assessed by the standard seedbox screening test (SSST) and modified bulk seeding test (MBST). Furthermore, three NILs (NIL-*BPH9*, NIL-*BPH17*, and NIL-*BPH32*) were tested for antibiosis and antixenosis under different nitrogen treatments. Such information would provide evidence of the impact of nitrogen on BPH resistance genes and further reveal candidate BPH resistance genes for IPM programs.

## Materials and methods

### Plant materials

Taichung Native 1 (TN1), IR24, and twelve NILs with one or two BPH resistance genes were used in this study. Twelve NILs were initially obtained from the IRRI (Jena et al. 2017). IR24, the recurrent parent of the NILs, was obtained from the National Plant Genetic Resources Center, Taiwan Agricultural Research Institute, Taiwan (TARI). The susceptible control TN1 used for the SSST was obtained from Dr. Shu-Jen Wang, National Taiwan University. Seeds were sterilized with 2% NaOCl (CLOROX, California, United States) for 30 min in a shaker and further washed with distilled water for 10 min. The seeds were germinated on a moistened paper towel under dark conditions at 37 °C for 2 days.

### Environmental setting

In this study, all plants were fertilized with ammonium sulfate, single superphosphate, and potassium chloride (Taiwan Fertilizer Company, Taiwan) to supply nitrogen, phosphate, and potassium, respectively. For nitrogen application, equivalent amounts of nitrogen were added to reach 0 kg ha^− 1^ (denoted N0), 50 kg ha^− 1^ (denoted N50), 100 kg ha^− 1^ (denoted N100), and 200 kg ha^− 1^ (denoted N200). The amounts of phosphate and potassium added were equivalent to 50 kg ha^− 1^ and 60 kg ha^− 1^, respectively. Before planting, basal fertilizer was applied at 30% for nitrogen, 100% for phosphate, and 40% for potassium. Plants and *N. lugens* were grown in a walk-in chamber with a day/night temperature of 30 °C/25 °C and a 12-h light/12-h dark cycle.

### Insects

An *N. lugens* colony (biotype 1) was obtained from the Chiayi Agricultural Experimental Station, TARI. *N. lugens* was mass-reared on TN1 seedlings in an insect cage (BugBorm-4, Megaview, Taichung, Taiwan) in a walk-in chamber with a day/night temperature of 30 °C/25 °C and a 12-h light/ 12-h dark cycle (L/D).

### SPAD value

The leaf chlorophyll contents of the three NILs (NIL-*BPH9*, NIL-*BPH17*, and NIL-*BPH32*) and IR24 were measured by a Soil Plant Analysis Development chlorophyll meter (SPAD 502 Plus Chlorophyll Meter 2900P, Konica Minolta, Osaka, Japan).

The SPAD is the alternative approach to measure the chlorophyll content without damaging leaf tissues. The average readings from the tip, middle, and base of the youngest expanded leaf of a 30-day-old rice plant were calculated. Each treatment included five replicate plants, and the experiment was repeated three times.

### Standard seedbox screening test (SSST) and modified bulk seeding test (MBST)

Twelve NILs, IR24, and the susceptible control TN1 treated with different nitrogen applications (N0, N50, and N200) were evaluated for insect resistance by the SSST and MBST. Briefly, 24 seeds of each tested NIL/variety were sown in a row, and 20 seedlings were selected for the test. Fourteen days after sowing, 2nd- to 3rd-instar *N. lugens* were applied to the seedlings (8–10 *N. lugens* per seedling). For the SSST, the damage level was measured according to the standard evaluation system (IRRI 2013) when the susceptible control TN1 was dead. For the MBST, the seedling survival evaluation scale followed Jena et al. (2006). These experiments were repeated three times.

### Population growth rate (PGR), honeydew excretion, fecundity, egg hatchability, survival rate, and settlement preference of *N.lugens*

Quantification of the PGR, honeydew excretion, fecundity, egg hatchability, survival rate, and settlement preference was performed using methods previously described by Kuang et al. (2021). Briefly, germinated seeds of three NILs (NIL-*BPH9*, NIL-*BPH17*, and NIL-*BPH32*) and IR24 were transferred to 150 ml glass beakers containing Kimura B solution (Yoshida et al. 1971). After seven days, the seedlings were transferred into plastic pots (one plant per pot) with paddy soil treated with basal fertilizer application. Three nitrogen applications (N50, N100, and N200) were applied in these experiments. At 30 days after germination, all branches except the main tiller were removed. The sample size (n) and the number of replicates (N) in most of the assays were N = 3 and n = 5, respectively; however, in the honeydew excretion assay, these values were N = 4 and n = 5, and in the settlement preference test, these values were n = 500.

### Statistical analysis

All the data were analyzed using R software (v 4.0.5) (Team 2013). The SSST and MBST results were analyzed by two-way ANOVA, and the PGR, honeydew excretion, fecundity, egg hatchability, and survival rate data were analyzed by one-way ANOVA. The least significant difference test was used to detect differences at p < 0.05. In the multiple comparison procedure, Bonferroni’s correction method was applied to control the familywise error rate (FWER) to ensure a lower value than the nominal level of 0.05. The settlement preference data were analyzed using the standard z-test to evaluate whether or not *N. lugens* nymphs had settlement preference. Specifically, if they had no preference and selected the plants randomly, then the proportions of choosing IR24 and NIL-*BPH9/17/32* would be equal to 0.5.

## Results

### SSST with different nitrogen treatments

Twelve NILs and their recurrent parent IR24 were evaluated for the impact of different nitrogen treatments on resistance against *N. lugens* using an SSST. The damage scores of the experimental plants were affected by the variety and treatment x variety interaction (*p* value < 0.001; Table 1). Under the no-nitrogen regime (N0), 9 NILs (NIL-*BPH4*, NIL-*BPH9*, NIL-*BPH17*, NIL-*BPH20*, NIL-*BPH26*, NIL-*BPH32*, NIL-*BPH2 + 32*, NIL-*BPH9 + 32*, and NIL-*BPH18 + 32*) had a lower damage score than IR24, whereas 3 NILs (NIL-*BPH10*, NIL-*BPH18*, and NIL-*BPH21*) had similar scores to IR24 (Table 2). Under the low-nitrogen regime (N50), 9 NILs (NIL-*BPH4*, NIL-*BPH9*, NIL-*BPH17*, NIL-*BPH18*, NIL-*BPH20*, NIL-*BPH32*, NIL-*BPH2 + 32*, NIL-*BPH9 + 32*, and NIL-*BPH18 + 32*) had a lower damage score than IR24, whereas 3 NILs (NIL-*BPH10*, NIL-*BPH21*, and NIL-*BPH26*) had similar scores to IR24 (Table 2). Under the high-nitrogen regime (N200), 8 NILs (NIL-*BPH9*, NIL-*BPH17*, NIL-*BPH20*, NIL-*BPH21*, NIL-*BPH32*, NIL-*BPH2 + 32*, NIL-*BPH9 + 32*, and NIL-*BPH18 + 32*) had a lower damage score than IR24, whereas 3 NILs (NIL-*BPH4*, NIL-*BPH10*, and NIL-*BPH18*) had similar scores to IR24 (Table 2). In addition, NIL-*BPH26* had a higher damage score than IR24 under N200. Compared with the N0 and N200 regimes, one NIL (NIL-*BPH21*) and IR24 showed variation in their resistance levels (Table 2). IR24 and NIL-*BPH21* showed no resistance under N0 and N50 but gained resistance under N200. Overall, 4 NILs carrying a single BPH resistance gene (NIL-*BPH9*, NIL-*BPH17*, NIL-*BPH20*, and NIL-*BPH32*) and 3 NILs with gene pyramiding (NIL-*BPH2 + 32*, NIL-*BPH9 + 32*, and NIL-*BPH18 + 32*) maintained their resistance under different nitrogen treatments.

**Table 1:** Two-way ANOVA of the SSST results of NIL responses to factors

Source of variation	df	*F* value	*p* value
Treatment^a^	2	0.4887	0.6148^ns^
Variety^b^	13	24.4085	< 0.0001***
Treatment × variety	26	2.6875	0.0002***
Residuals	102		

^a^N0, N50, N200

^b^TN1, IR24, NIL-*BPH4*, NIL-*BPH9*, NIL-*BPH10*, NIL-*BPH17*, NIL-*BPH18*, NIL-*BPH20*, NIL-*BPH21*, NIL-*BPH26*, NIL-*BPH32*, NIL-*BPH2 + 32*, NIL-*BPH18 + 32*, NIL-*BPH9 + 32*

^*ns* ^no significance, ***p value < 0.001

**Table 2:** SSST of NILs under different nitrogen regimes

Varieties/NILs	TN1	IR24	NIL-*BPH 4*	NIL-*BPH 9*	NIL-*BPH 10*	NIL-*BPH 17*	NIL-*BPH 18*	NIL-*BPH 20*	NIL-*BPH 21*	NIL-*BPH 26*	NIL-*BPH 32*	NIL-*BPH 2 + 32*	NIL-*BPH 9 + 32*	NIL-*BPH 18 + 32*
Nitrogen regimes														
N0	9.00 ± 0.00 a	7.67 ± 0.26 b	6.67 ± 0.29 cdef	5.50 ± 0.87 ghij	7.83 ± 0.76 b	3.00 ± 0.00 n	7.50 ± 0.50 bc	5.83 ± 1.61 fghi	7.00 ± 1.00 bcde	6.67 ± 0.76 cdef	5.67 ± 1.15 fghij	5.33 ± 0.58 hijk	4.33 ± 0.58 klm	4.67 ± 0.58 jklm
N50	9.00 ± 0.00 a	7.58 ± 0.74 b	6.17 ± 0.29 efgh	4.33 ± 0.58 klm	7.50 ± 0.87 bc	3.67 ± 0.58 mn	6.67 ± 0.76 cdef	6.50 ± 0.50 defg	7.33 ± 0.76 bcd	7.5 ± 0.87 bc	5.00 ± 0.00 ijkl	5.67 ± 0.57 fghij	3.67 ± 0.58 mn	5.00 ± 1.00 ijkl
N200	9.00 ± 0.00 a	6.58 ± 0.58 def	6.33 ± 1.15 defgh	5.33 ± 0.58 hijk	7.33 ± 0.58 bcd	4.00 ± 0.00 lmn	6.67 ± 0.58 cdef	5.00 ± 0.00 ijkl	4.67 ± 0.58 jklm	7.5 ± 0.87 bc	4.67 ± 0.58 jklm	4.33 ± 0.58 klm	4.33 ± 0.58 klm	4.00 ± 0.00 lmn

The damage score of *N. lugens* nymphs feeding on TN1, IR24, and NILs was determined using the standard evaluation method (IRRI 2013). Means followed by different letters are significantly different (*p* < 0.05)

### MBST with different nitrogen treatments

The resistance score of the tested plants was affected by the variety and treatment x variety interaction (*p* value < 0.001; Table 3). Under the no-nitrogen treatment (N0), 7 NILs (NIL-*BPH9*, NIL-*BPH17*, NIL-*BPH20*, NIL-*BPH32*, NIL-*BPH2 + 32*, NIL-*BPH9 + 32*, and NIL-*BPH18 + 32*) had a higher resistance score than IR24, whereas 5 NILs (NIL-*BPH4*, NIL-*BPH10*, NIL-*BPH18*, NIL-*BPH21*, and NIL-*BPH26*) had a lower survival rate similar to IR24 (Table 4). Under the low-nitrogen treatment (N50), 7 NILs (NIL-*BPH4*, NIL-*BPH9*, NIL-*BPH17*, NIL-*BPH32*, NIL-*BPH2 + 32*, NIL-*BPH9 + 32*, and NIL-*BPH18 + 32*) had a higher resistance score than IR24, and 5 NILs (NIL-*BPH10*, NIL-*BPH18*, NIL-*BPH20*, NIL-*BPH21*, and NIL-*BPH26*) had a lower survival rate similar to IR24 (Table 4). Under the high-nitrogen treatment (N200), 8 NILs (NIL-*BPH4*, NIL-*BPH17*, NIL-*BPH20*, NIL-*BPH21*, NIL-*BPH32*, NIL-*BPH2 + 32*, NIL-*BPH9 + 32*, and NIL-*BPH18 + 32*) had a higher resistance score than IR24, whereas 4 NILs (NIL-*BPH9*, NIL-*BPH10*, NIL-*BPH18*, and NIL-*BPH26*) had a lower survival rate similar to IR24 (Table 4). Compared with the N0 and N200 regimes, two NILs (NIL-*BPH4* and NIL-*BPH21*) showed variation in their resistance levels (Table 4). NIL-*BPH4* and NIL-*BPH21* showed no resistance under the N0 and N50 regimes but gained resistance under the N200 regime. Overall, 2 NILs carrying a single BPH resistance gene (NIL-*BPH17* and NIL-*BPH32*) and 3 NILs with gene pyramiding (NIL-*BPH2 + 32*, NIL-*BPH9 + 32*, and NIL-*BPH18 + 32*) maintained their resistance under nitrogen treatments.

**Table 3:** Two-way ANOVA of the MBST results of NIL responses to factors

	df	*F* value	*p* value
Treatment^a^	2	0.6889	0.5045^ns^
Variety^b^	13	18.5636	< 0.0001***
Treatment × variety	26	3.2536	< 0.0001***
Residuals	102		

^a^N0, N50, N200

^b^TN1, IR24, NIL-*BPH4*, NIL-*BPH9*, NIL-*BPH10*, NIL-*BPH17*, NIL-*BPH18*, NIL-*BPH20*, NIL-*BPH21*, NIL-*BPH26*, NIL-*BPH32*, NIL-*BPH2 + 32*, NIL-*BPH18 + 32*, NIL-*BPH9 + 32*

^*ns* ^no significance, ***p value < 0.001

**Table 4:** MBST of NILs under different nitrogen regimes

Varieties/NILs	TN1	IR24	NIL-*BPH 4*	NIL-*BPH 9*	NIL-*BPH 10*	NIL-*BPH 17*	NIL-*BPH 18*	NIL-*BPH 20*	NIL-*BPH 21*	NIL-*BPH 26*	NIL-*BPH 32*	NIL-*BPH 2 + 32*	NIL-*BPH 9 + 32*	NIL-*BPH 18 + 32*
Nitrogen regimes														
N0	8.3 ± 0.5 a	6.0 ± 0.0 cd	5.0 ± 0.0 def	4.0 ± 1.0 efg	6.7 ± 0.6 bc	0.0 ± 0.0 l	5.7 ± 0.6 cde	4.0 ± 2.6 efg	5.3 ± 1.2 cdef	5.0 ± 1.0 def	4.0 ± 1.0 efg	2.7 ± 1.2 ghi	0.3 ± 0.6 kl	1.7 ± 0.6 ijkl
N50	8.5 ± 0.5 a	6.5 ± 1.2 c	4.7 ± 0.6 def	0.7 ± 1.2 jkl	6.0 ± 1.0 cd	0.3 ± 0.6 kl	5.3 ± 0.6 cdef	5.3 ± 0.6 cdef	6.0 ± 1.0 cd	6.0 ± 1.0 cd	2.0 ± 0.0 hijk	4.0 ± 1.7 efg	0.3 ± 0.6 kl	2.7 ± 1.5 ghi
N200	8.0 ± 1.1 ab	5.2 ± 0.8 def	2.7 ± 2.1 ghi	5.0 ± 1.0 def	5.7 ± 0.6 cde	0.3 ± 0.6 kl	3.7 ± 1.5 fgh	2.3 ± 2.1 ghij	1.7 ± 0.6 ijkl	4.7 ± 2.3 def	2.0 ± 1.0 hijk	2.0 ± 1.7 hijk	0.3 ± 0.6 kl	0.0 ± 0.0 l

The survival rate score of *N. lugens* nymphs feeding on TN1, IR24, and NILs was based pm that reported by Jena et al. (2006). Means followed by different letters are significantly different (*p* < 0.05)

### Resistance of NIL-BPH9, NIL-BPH17, and NIL-BPH32 under nitrogen treatments

**Fig. 1:**
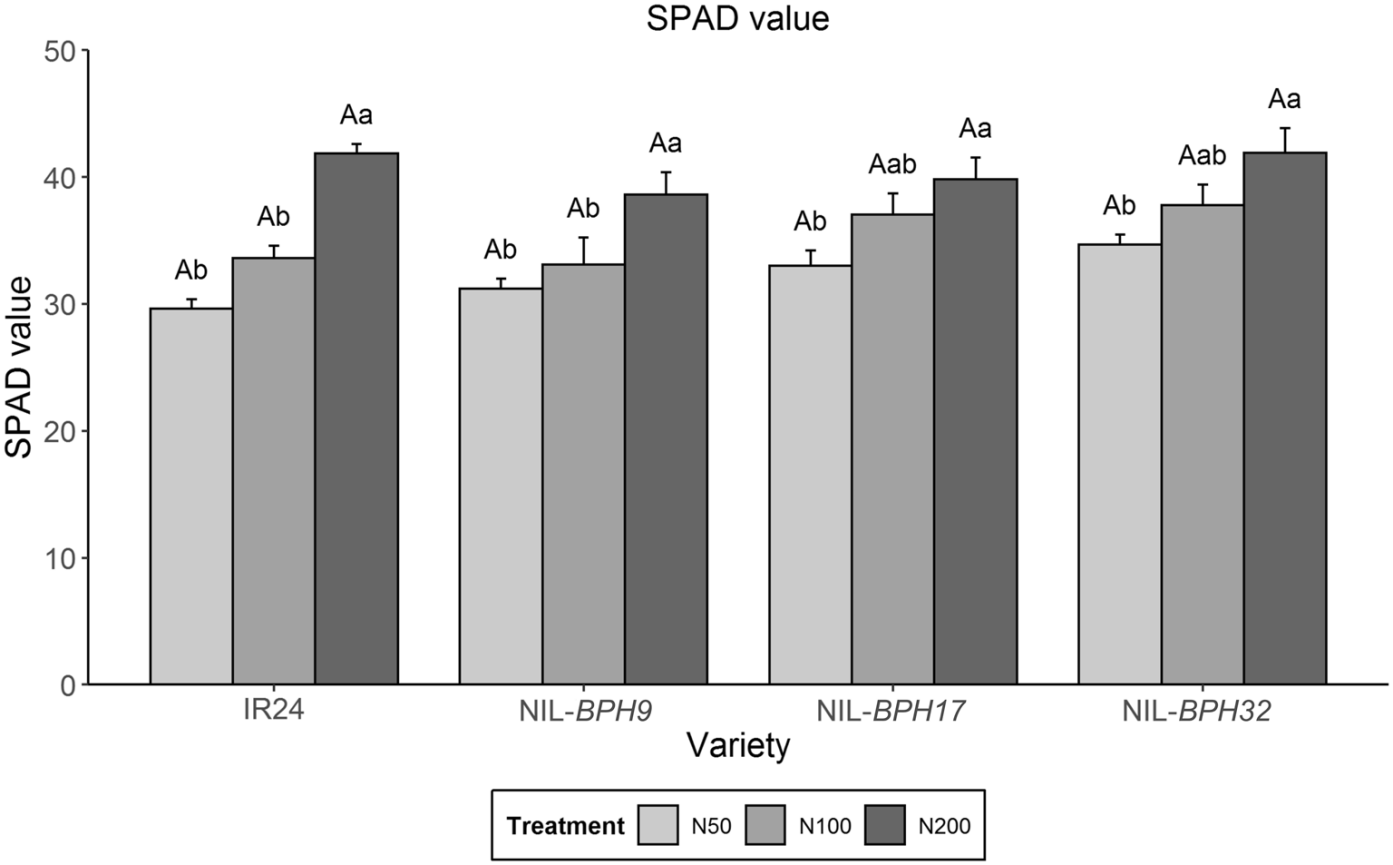
The SPAD values of IR24, NIL-*BPH9*, NIL-*BPH17*, and NIL-*BPH32* under different nitrogen regimes (N50, N100, and N200). Means in each column followed by the same capital letter do not differ significantly among varieties (*p* < 0.05). Means in each column followed by the same lowercase letter do not differ significantly among different nitrogen treatments. The error bars indicate the S.Es

Based on the above data, three NILs (NIL-*BPH9*, NIL-*BPH17*, and NIL-*BPH32*) were selected to test for antibiosis and antixenosis effects under different nitrogen applications. The PGR, honeydew excretion, fecundity, egg hatchability, and survival rate were used to test for antibiosis effects, while a settlement preference test was used to test for an antixenosis effect. Nitrogen is the main factor affecting crop yield. No nitrogen application (N0) is not applicable in farming practices. Thus, three nitrogen treatments (N50, N100, and N200) were selected to study the effects further. The chlorophyll content of NIL-*BPH9*, NIL-*BPH17*, and NIL-*BPH32* under the nitrogen treatments was measured. The SPAD value was not different among the tested plants with the same nitrogen treatment (Fig. 1). However, all tested varieties had higher SPAD values under the N200 treatment than under the other two nitrogen regimes (N50 and N100), except NIL-*BPH17* and NIL-*BPH32* under the N100 regime (Fig. 1).

**Fig. 2:**
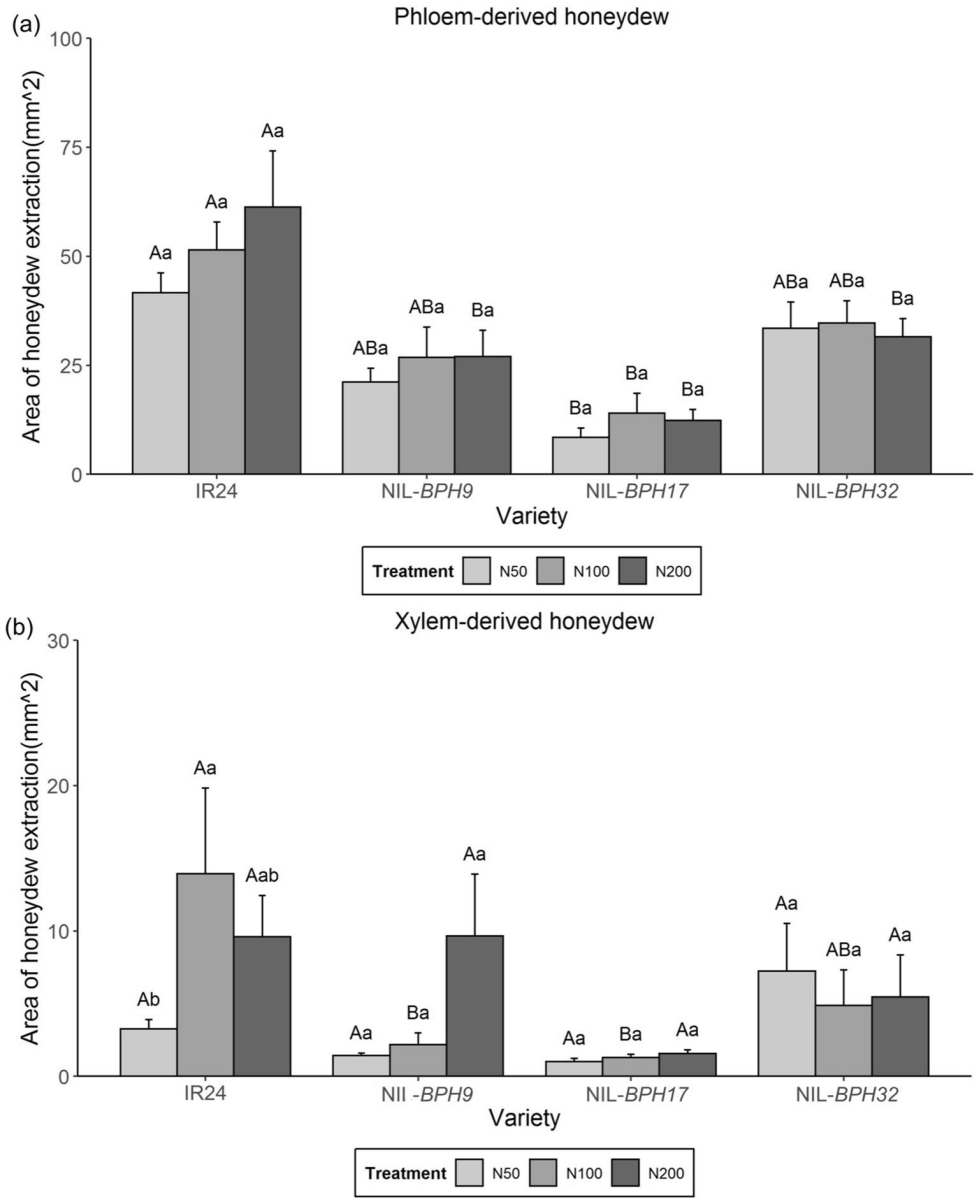
Areas of honeydew excretion of *N. lugens* females feeding on IR24, NIL-*BPH9*, NIL-*BPH17*, and NIL-*BPH32* under different nitrogen regimes. **a** Phloem-derived excretion. **b** Xylem-derived excretion. Means in each column followed by the same capital letter do not differ significantly among varieties (*p* < 0.05). Means in each column followed by the same lowercase letter do not differ significantly among different nitrogen treatments. The error bars indicate the S.Es

A honeydew excretion assay was implemented as an indirect method to examine the phloem and xylem sap consumption of *N. lugens*. For phloem-derived honeydew, *N. lugens* feeding on NIL-*BPH17* had lower phloem sap consumption than *N. lugens* feeding on IR24 under all nitrogen treatments, while *N. lugens* feeding on NIL-*BPH9* and NIL-*BPH32* had lower phloem sap consumption than *N. lugens* feeding on IR24 under the N200 treatment (Fig. 2a). Among the nitrogen treatments, no difference was found among *N. lugens* feeding on NIL-*BPH9*, NIL-*BPH17*, and NIL-*BPH32* (Fig. 2a). For xylem-derived honeydew, *N. lugens* feeding on NIL-*BPH9* and NIL-*BPH17* had a lower xylem sap consumption than *N. lugens* feeding on IR24 under the N100 treatment (Fig. 2b). *N. lugens* feeding on IR24 under the N100 treatment had higher xylem sap consumption than that under the N50 treatment, whereas no difference was found among *N. lugens* feeding on NIL-*BPH9*, NIL-*BPH17*, and NIL-*BPH32* (Fig. 2b).

**Fig. 3:**
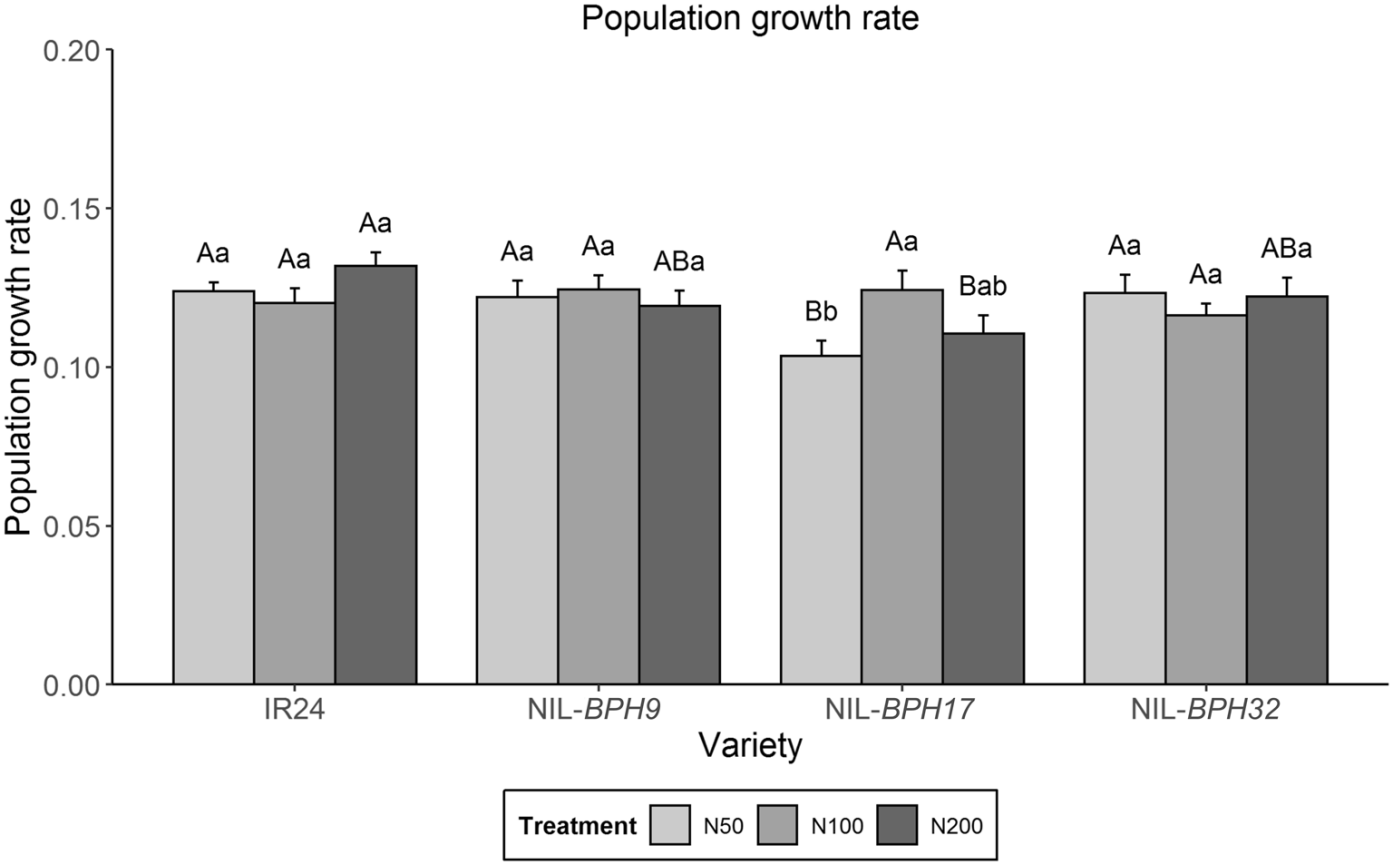
Population growth rate of *N. lugens* nymphs feeding on IR24, NIL-*BPH9*, NIL-*BPH17*, and NIL-*BPH32* under different nitrogen regimes. Means in each column followed by the same capital letter do not differ significantly among varieties (*p* < 0.05). Means in each column followed by the same lowercase letter do not differ significantly among different nitrogen treatments. The error bars indicate the S.Es

**Fig. 4:**
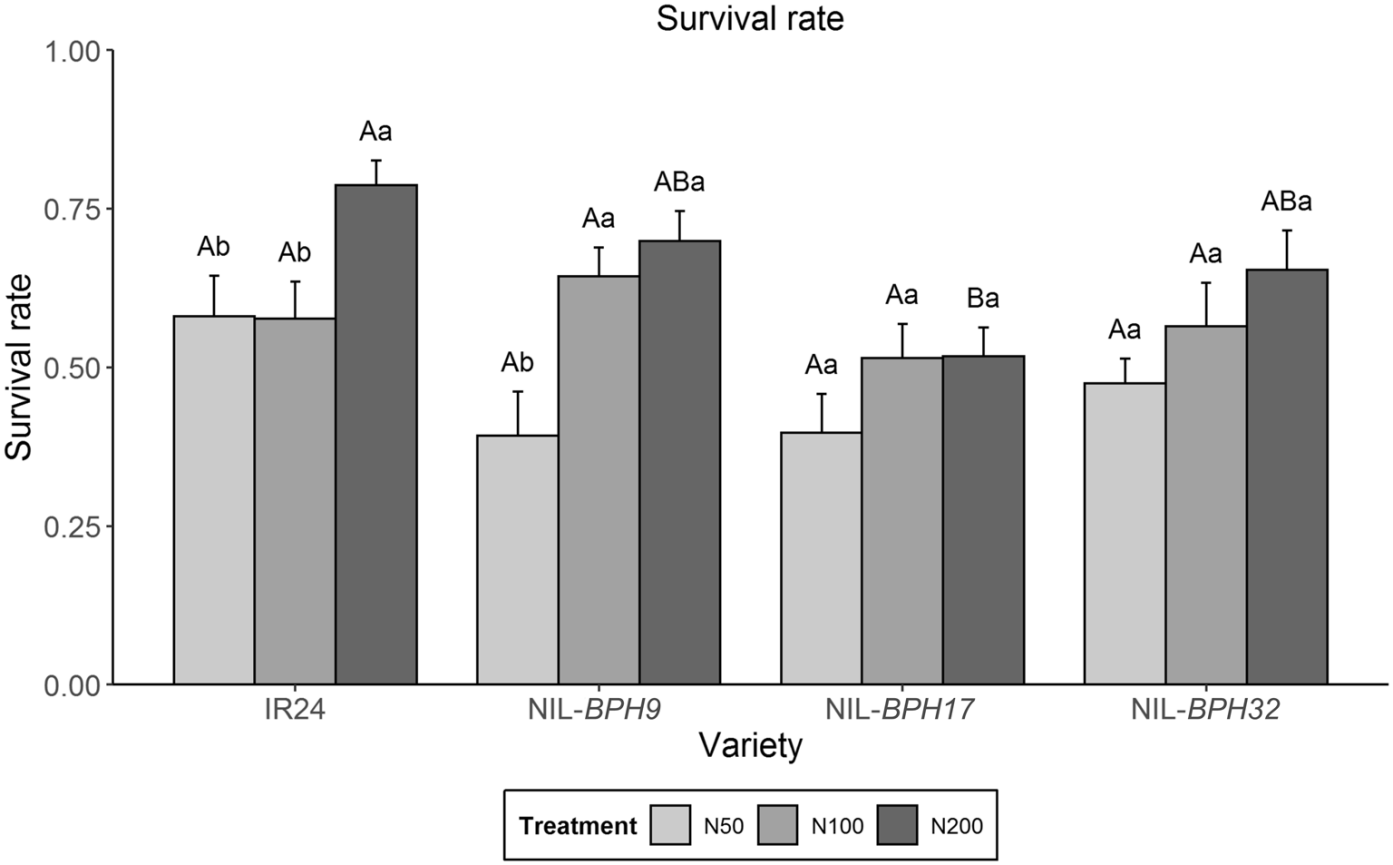
Nymph survival rate of *N. lugens* feeding on IR24, NIL-*BPH9*, NIL-*BPH17*, and NIL-*BPH32* under different nitrogen regimes. Means in each column followed by the same capital letter do not differ significantly among varieties (*p* < 0.05). Means in each column followed by the same lowercase letter do not differ significantly among different nitrogen treatments. The error bars indicate the S.Es

The PGR was used as the growth parameter of *N. lugens*. There was no difference between IR24 and NIL-*BPH9* or NIL-*BPH32* under any nitrogen treatments (Fig. 3). However, *N. lugens* had a lower PGR on NIL-*BPH17* than on IR24 under the N50 and N200 treatments but similar PGRs on NIL-*BPH17* and IR24 under the N100 treatment (Fig. 3). In addition, *N. lugens* feeding on NIL-*BPH17* had a lower PGR under the N50 and treatment than under the N100 treatment, whereas no difference was found among IR24, NIL-*BPH9*, and NIL-*BPH32* under any treatment (Fig. 3). The nymph survival rate of *N. lugens* on these NILs under different nitrogen treatments was further examined. *N. lugens* nymphs on NIL-*BPH17* had a lower survival rate than those on IR24 under the N200 treatment, whereas no difference was found among IR24, NIL-*BPH9*, and NIL-*BPH32* under any of the treatments (Fig. 4). Within the same variety, *N. lugens* nymphs feeding on IR24 under the N200 regime had a higher survival rate than those feeding on IR24 under low nitrogen application (N50 and N100 treatments), whereas there was no difference with NIL-*BPH17* and NIL-*BPH32* (Fig. 4).

**Fig. 5:**
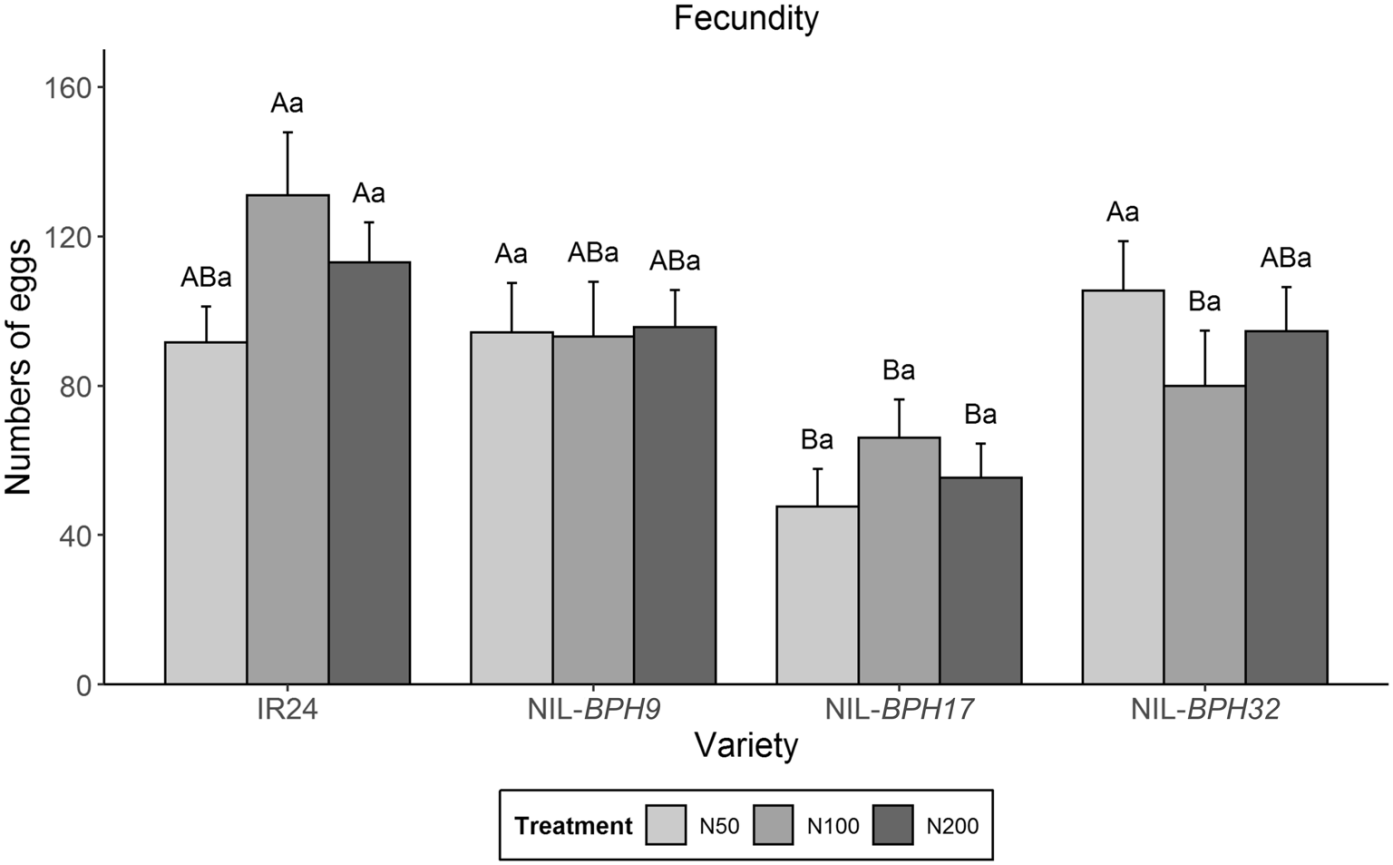
* N. lugens* female fecundity on IR24, NIL-*BPH9*, NIL-*BPH17*, and NIL-*BPH32* under different nitrogen regimes. Means in each column followed by the same capital letter do not differ significantly among varieties (*p* < 0.05). Means in each column followed by the same lowercase letter do not differ significantly among different nitrogen treatments. The error bars indicate the S.Es

**Fig. 6:**
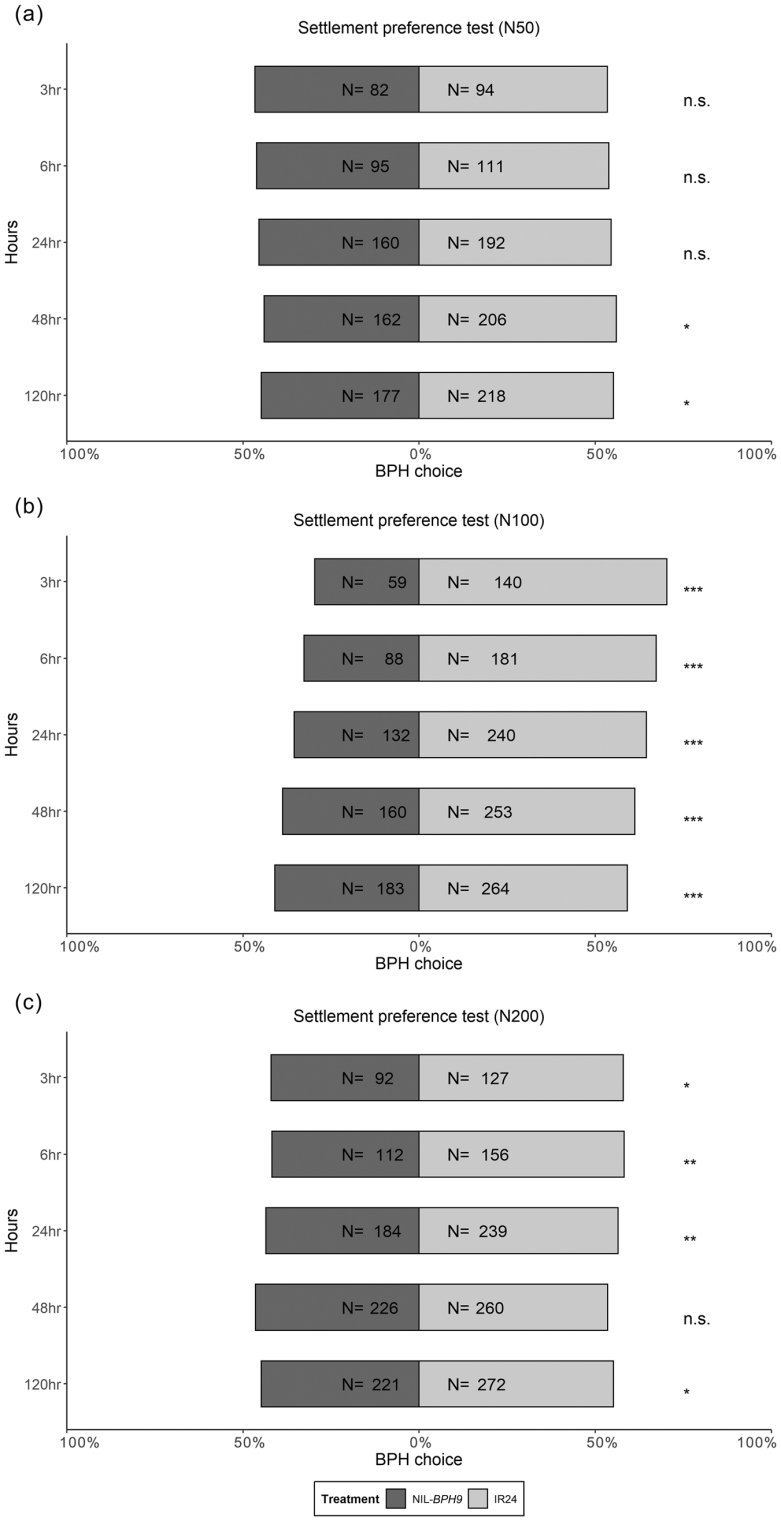
Choice test of *N. lugens* nymphs on IR24 and NIL-*BPH9* under different nitrogen regimes. **a** N50. **b** N100. **c** N200. The asterisks indicate differences between IR24 and NIL-*BPH9* as *p < 0.05; **p < 0.01; ***p < 0.001; *ns* no significance

**Fig. 7:**
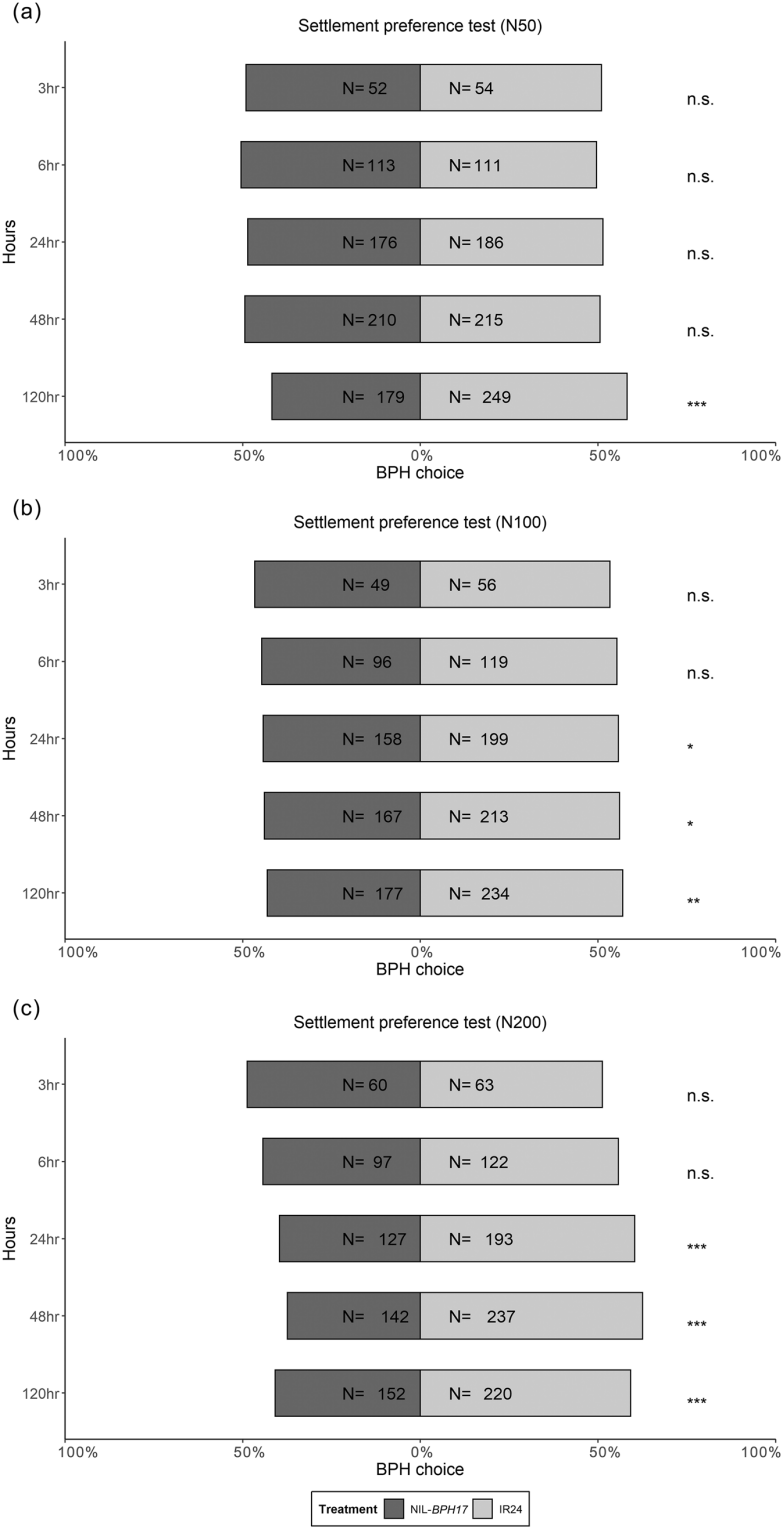
Choice test of *N. lugens* nymphs on IR24 and NIL-*BPH17* under different nitrogen regimes. **a** N50. **b** N100. **c** N200. The asterisks indicate differences between IR24 and NIL-*BPH17* as *p < 0.05; **p < 0.01; ***p < 0.001; *ns* no significance

**Fig. 8:**
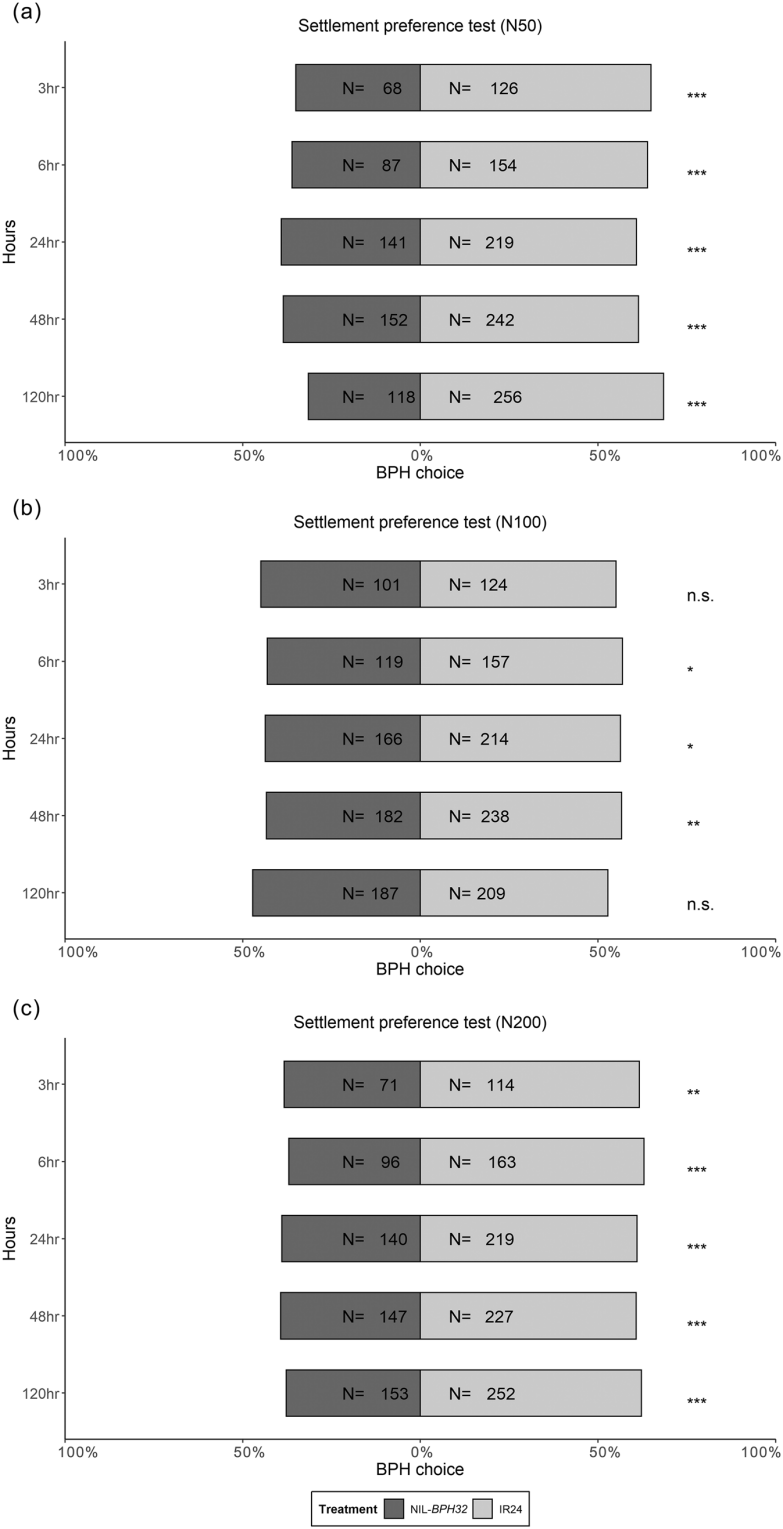
Choice test of *N. lugens* nymphs on IR24 and NIL-*BPH32* under different nitrogen regimes. **a** N50. **b** N100. **c** N200. The asterisks indicate differences between IR24 and NIL-*BPH32* as *p < 0.05; **p < 0.01; ***p < 0.001; *ns* no significance

Using a no-choice assay, female fecundity under different nitrogen applications was examined. *N. lugens* female adults on NIL-*BPH32* had lower fecundity than those on IR24 under the N100 treatment, whereas *N. lugens* female adults on NIL-*BPH17* had lower fecundity than those on IR24 under all nitrogen treatments (Fig. 5). In the settlement preference test, *N. lugens* nymphs preferred settling on IR24 over NIL-*BPH9* from 48 to 120 h under the N50 treatment (Fig. 6a). Under the N100 and N200 treatments, *N. lugens* nymphs preferred IR24 from 3 to 120 h, except at the 48-h time point under the N200 treatment (Fig. 6b, c). When comparing IR24 and NIL-*BPH17*, *N. lugens* nymphs preferred IR24 at 120 h under the N50 treatment and preferred IR24 at 24 h under the N100 and N200 treatments (Fig. 7). For comparing IR24 and NIL-*BPH32*, *N. lugens* nymphs preferred IR24 from 3 to 120 h under all nitrogen treatments except the 3 and 120 h time points under the N100 treatment (Fig. 8).

## Discussion

Plants with insect resistance traits are keys to IPM programs. Since BPH-resistant rice varieties have been used in the market, the environmental impact on their resistance has been noticed (Horgan et al. 2021; Kuang et al. 2021). Nitrogen input is highly correlated with crop yield. However, excessive nitrogen input may not increase crop production but instead benefit insect pests. Therefore, the amount of nitrogen input should be appropriate and considered in the IPM program. In this study, we examined twelve NILs with BPH resistance genes under different nitrogen regimes. Three NILs (NIL-*BPH9*, NIL-*BPH17*, and NIL-*BPH32*) maintained a low damage score under varying nitrogen applications. High nitrogen input would increase the SPAD value, the indicator of chlorophyll content, in the leaf tissues. Based on the *N. lugens* growth parameters, the resistance of the three tested NILs did not respond to different nitrogen regimes, whereas NIL-*BPH17* exerted the strongest inhibitory effect on *N. lugens* growth and development.

The fitness of *N. lugens* increases with increases in the plant nitrogen content in rice (Lu et al. 2004; Rashid et al. 2016, 2017a, b). Increasing the soil nitrogen level increases the survival rate and weight of *N. lugens* nymphs and shortens their developmental period (Horgan et al. 2016, 2018; Rashid et al. 2017b). Some of these results were consistent with those of our study (Fig. 4). In addition, the biomass of *N. lugens* nymphs on a susceptible variety (T65) increased under a high-nitrogen regime (Srinivasan et al. 2015). The impact of nitrogen on the fitness of *N. lugens* adults is not conclusive. The adult longevity, fecundity, hatchability, weight, and survival rate of *N. lugens* increases with increases in the nitrogen content of the host plants (Rashid et al. 2016, 2017b). However, Horgan et al. (2016) reported that nitrogen fertilizer treatments did not affect fecundity or egg mortality. Our study supports the last finding. In addition, under a high-nitrogen regime, *N. lugens* fecundity increased across generations (Lu et al. 2004). This study revealed that *N. lugens* outbreaks may frequently occur in nitrogen-enriched crops (Lu et al. 2004; Horgan et al. 2021) reported that increasing nitrogen input would reduce resistance in rice but enhance its tolerance to *N. lugens*. Our study yielded similar results on IR24 (Table 2). However, in our study, *N. lugens* feeding on three tested NILs (NIL-*BPH9*, NIL-*BPH17*, and NIL-*BPH32*) under high nitrogen input did not overcome the resistance. These results indicated that these three BPH resistance genes would benefit rice breeding programs.

Breeding insect-resistant varieties with insect resistance genes is an effective and environmentally friendly strategy for IPM programs. Several technologies, including marker-assisted selection and gene editing, accelerate the breeding process. BPH resistance genes have been developed in several rice varieties, such as 9311, IR24, and T65 (Jena et al. 2017; Nguyen et al. 2019; Xiao et al. 2016). Furthermore, NILs with resistance to other phloem feeders, including the white-backed planthopper (*S. furcifera*), gall midge (*Orseolia oryzae*), and green rice leafhopper (*N. cincticeps*), have also been developed (Fujita et al. 2010; Himabindu et al. 2010; Yamasaki et al. 2003). However, because *N. lugens* has multiple biotypes and is prone to adaptation, rice varieties with a single resistance gene may show a reduction in resistance within a few years (Jena and Kim 2010). Furthermore, several BPH resistance genes lose their efficacy under environmental changes (Kuang et al. 2021). Thus, pyramiding multiple genes would be a better strategy. It has been reported that pyramided genes have a synergistic effect (Hu et al. 2013; Jena et al. 2017; Qiu et al. 2012). In our study, under N0 treatment, NIL-*BPH9 + 32* and NIL-*BPH18 + 32* had lower damage scores in the SSST and higher resistance in the MBST than the NILs with a single resistance gene (*BPH9*, *BPH18*, and *BPH32*). Furthermore, NIL-*BPH9 + 32* and NIL-*BPH18 + 32* showed a similar trend under environmental change (Kuang et al. 2021). Thus, pyramiding genes in one variety not only prevents the loss of efficacy but also enhances resistance to environmental changes, including climate change and varying nitrogen inputs.

Climate change impact and excessive nitrogen input are the two critical challenges to our crop production. Therefore, we would like to use this unique NIL set to find out BPH genes that would maintain the resistance under stress. Based on the findings of this study and our previous results, NIL-*BPH17* maintained resistance against *N. lugens* under not only environmental changes (high atmospheric temperature and high CO_2_ concentration) but also varying nitrogen applications (Kuang et al. 2021). Furthermore, our results showed that increasing the nitrogen level enhanced the preferences of *N. lugens* for IR24 from 120 h to 24 h after the experiment (Fig. 7). In addition, with environmental changes, the preferences of *N. lugens* nymphs for IR24 and NIL-*BPH17* accelerated from 24 h to 6 h after the experiment (Kuang et al. 2021). Thus, *BPH17* may be the best BPH resistance gene for insect resistance breeding programs in rice.

## Conclusions

The impact of nitrogen on the resistance of twelve NILs with BPH resistance genes against *N. lugens* was examined. Nitrogen input affected some of the tested lines with BPH resistance genes. However, three NILs (NIL-*BPH9*, NIL-*BPH17*, and NIL-*BPH32*) did not show changes in resistance with different nitrogen regimes, while NIL-*BPH17* had the strongest inhibitory effect on *N. lugens* growth and development. These results provide valuable information for IPM programs.

## Data Availability

The data used and analyzed for the current study can be obtained from the corresponding author.

## References

[CR1] Awmack CS, Leather SR (2002). Host plant quality and fecundity in herbivorous insects. Ann Rev Entomol.

[CR2] Bhardwaj U, Bhardwaj A, Kumar R, Leelavathi S, Reddy VS, Mazumdar-Leighton S (2014). Revisiting Rubisco as a protein substrate for insect midgut proteases. Arch Insect Biochem Physiol.

[CR3] Cheng X, Wu Y, Guo J, Du B, Chen R, Zhu L, He G (2013). A rice lectin receptor-like kinase that is involved in innate immune responses also contributes to seed germination. Plant J.

[CR4] Cohen MB, Alam SN, Medina EB, Bernal CC (1997). Brown planthopper, *Nilaparvata*
*lugens*, resistance in rice cultivar IR64: mechanism and role in successful *N. lugens* management in Central Luzon, Philippines. Entomol Exp Appl.

[CR5] Du B, Zhang W, Liu B, Hu J, Wei Z, Shi Z, He R, Zhu L, Chen R, Han B, He G (2009). Identification and characterization of *Bph14*, a gene conferring resistance to brown planthopper in rice. Proc Natl Acad Sci USA.

[CR6] Du B, Chen R, Guo J, He G (2020). Current understanding of the genomic, genetic, and molecular control of insect resistance in rice. Mol Breeding.

[CR7] Fujita D, Yoshimura A, Yasui H (2010). Development of near-isogenic lines and pyramided lines carrying resistance genes to green rice leafhopper (*Nephotettix cincticeps* Uhler) with the Taichung 65 genetic background in rice (*Oryza sativa* L.). Breed Sci.

[CR8] Guo J, Xu C, Wu D, Zhao Y, Qiu Y, Wang X, Ouyang Y, Cai B, Liu X, Jing S (2018). *Bph6* encodes an exocyst-localized protein and confers broad resistance to planthoppers in rice. Nat Genet.

[CR9] Himabindu K, Suneetha K, Sama V, Bentur J (2010). A new rice gall midge resistance gene in the breeding line CR57-MR1523, mapping with flanking markers and development of NILs. Euphytica.

[CR10] Horgan FG, Srinivasan TS, Naik BS, Ramal AF, Bernal CC, Almazan MLP (2016). Effects of nitrogen on egg-laying inhibition and ovicidal response in planthopper-resistant rice varieties. Crop Prot.

[CR11] Horgan FG, Cruz AP, Bernal CC, Ramal AF, Almazan MLP, Wilby A (2018). Resistance and tolerance to the brown planthopper, *Nilaparvata lugens* (Stål), in rice infested at different growth stages across a gradient of nitrogen applications. Field Crops Res.

[CR12] Horgan FG, de Freitas TFS, Crisol-Martínez E, Mundaca EA, Bernal CC (2021). Nitrogenous fertilizer reduces resistance but enhances tolerance to the brown planthopper in fast-growing, moderately resistant rice. Insects.

[CR13] Hu J, Cheng M, Gao G, Zhang Q, Xiao J, He Y (2013). Pyramiding and evaluation of three dominant brown planthopper resistance genes in the elite indica rice 9311 and its hybrids. Pest Manag Sci.

[CR14] IRRI (2013). Standard evaluation system for rice.

[CR15] Jena KK, Kim SM (2010). Current status of brown planthopper (BPH) resistance and genetics. Rice.

[CR16] Jena KK, Jeung JU, Lee JH, Choi HC, Brar DS (2006). High-resolution mapping of a new brown planthopper (BPH) resistance gene, *Bph18* (t), and marker-assisted selection for BPH resistance in rice (*Oryza sativa* L.). Theor Appl Genet.

[CR17] Jena KK, Hechanova SL, Verdeprado H, Prahalada GD, Kim S-R (2017). Development of 25 near-isogenic lines (NILs) with ten BPH resistance genes in rice (*Oryza sativa* L.): Production, resistance spectrum, and molecular analysis. Theor Appl Genet.

[CR18] Ji H, Kim SR, Kim YH, Suh JP, Park HM, Sreenivasulu N, Misra G, Kim SM, Hechanova SL, Kim H, Lee GS, Yoon UH, Kim TH, Lim H, Suh SC, Yang J, An G, Jena KK (2016). Map-based cloning and characterization of the *BPH18* gene from wild rice conferring resistance to brown planthopper (BPH) insect pest. Sci Rep.

[CR19] Jiang M, Cheng J (2003). Feeding, oviposition and survival of overwintered rice water weevil (Coleoptera: Curculionidae) adults in response to nitrogen fertilization of rice at seedling stage. Appl Entomol Zool.

[CR20] Jlibene M, Nsarellah N (2011). Wheat breeding in Morocco, a historical perspective. The world wheat book: history of wheat breeding.

[CR21] Kuang YH, Fang YF, Lin SC, Tsai SF, Yang ZW, Li CP, Huang SH, Hechanova SL, Jena KK, Chuang WP (2021). The impact of climate change on the resistance of rice near-isogenic lines with resistance genes against brown planthopper. Rice.

[CR22] Liu Y, Wu H, Chen H, Liu Y, He J, Kang H, Sun Z, Pan G, Wang Q, Hu J, Hu J, Zhou F, Zhou K, Zheng X, Ren Y, Chen L, Wang Y, Zhao Z, Lin Q, Wu F, Zhang X, Guo X, Cheng X, Jiang L, Wu C, Wang H, Wan J (2015). A gene cluster encoding lectin receptor kinases confers broad-spectrum and durable insect resistance in rice. Nat Biotechnol.

[CR23] Lu ZX, Heong KL (2009). Effects of nitrogen-enriched rice plants on ecological fitness of planthoppers. Planthoppers: new threats to the sustainability of intensive rice production systems in Asia.

[CR24] Lu ZX, Heong KL, Yu XP, Hu C (2004). Effects of plant nitrogen on ecological fitness of the brown planthopper, *Nilaparvata lugens* Stal. in rice. J Asia Pac Entomol.

[CR25] Nguyen CD, Verdeprado H, Zita D, Sanada-Morimura S, Matsumura M, Virk PS, Brar DS, Horgan FG, Yasui H, Fujita D (2019). The development and characterization of near-isogenic and pyramided lines carrying resistance genes to brown planthopper with the genetic background of japonica rice (*Oryza sativa* L.). Plants.

[CR26] Nsarellah N, Amri A, Nachit M, Bouhssini ME, Lhaloui S, Lorenzoni C (2003). New durum wheat with Hessian fly resistance from *Triticum araraticum* and *T. carthlicum* in Morocco. Plant Breeding.

[CR27] Painter RH (1951). Insect resistance in crop plants.

[CR28] Peñalver Cruz A, Arida A, Heong KL, Horgan FG (2011). Aspects of brown planthopper adaptation to resistant rice varieties with the *Bph3* gene. Entomol Exp Appl.

[CR29] Prestidge R (1982). Instar duration, adult consumption, oviposition and nitrogen utilization efficiencies of leafhoppers feeding on different quality food (Auchenorrhyncha: Homoptera). Ecol Entomol.

[CR30] Qiu Y, Guo J, Jing S, Zhu L, He G (2012). Development and characterization of japonica rice lines carrying the brown planthopper-resistance genes *BPH12* and *BPH6*. Theor Appl Genet.

[CR31] Rashid M, Jahan M, Islam K (2016). Response of adult brown planthopper *Nilaparvata lugens* (Stål) to rice nutrient management. Neotrop Entomol.

[CR32] Rashid M, Ahmed N, Jahan M, Islam K, Nansen C, Willers J, Ali M (2017). Higher fertilizer inputs increase fitness traits of brown planthopper in rice. Sci Rep.

[CR33] Rashid M, Jahan M, Islam KS, Latif MA (2017). Ecological fitness of brown planthopper, *Nilaparvata lugens* (Stål), to rice nutrient management. Ecol Process.

[CR34] Ren J, Gao F, Wu X, Lu X, Zeng L, Lv J, Su X, Luo H, Ren G (2016). *Bph32*, a novel gene encoding an unknown SCR domain-containing protein, confers resistance against the brown planthopper in rice. Sci Rep.

[CR35] Smith CM (2005). Plant resistance to arthropods: molecular and conventional approaches.

[CR36] Srinivasan TS, Almazan MLP, Bernal CC, Fujita D, Ramal AF, Yasui H, Subbarayalu MK, Horgan FG (2015). Current utility of the *BPH25* and *BPH26* genes and possibilities for further resistance against plant-and leafhoppers from the donor cultivar ADR52. Appl Entomol Zool.

[CR37] Tamura Y, Hattori M, Yoshioka H, Yoshioka M, Takahashi A, Wu J, Sentoku N, Yasui H (2014). Map-based cloning and characterization of a brown planthopper resistance gene *BPH26* from *Oryza sativa* L. ssp. indica cultivar ADR52. Sci Rep.

[CR38] Team RC (2013). R: a language and environment for statistical computing.

[CR39] Visarto P, Zalucki MP, Nesbitt HJ, Jahn GC (2001). Effect of fertilizer, pesticide treatment, and plant variety on the realized fecundity and survival rates of brown planthopper, *Nilaparvata lugens* (Stål) (Homoptera: Delphacidae)—generating outbreaks in Cambodia. J Asia Pac Entomol.

[CR40] Wang Y, Cao L, Zhang Y, Cao C, Liu F, Huang F, Qiu Y, Li R, Lou X (2015). Map-based cloning and characterization of *BPH29*, a B3 domain-containing recessive gene conferring brown planthopper resistance in rice. J Exp Bot.

[CR41] Wier AT, Boethel DJ (1995). Feeding, growth, and survival of soybean looper (Lepidoptera: Noctuidae) in response to nitrogen fertilization of nonnodulating soybean. Environ Entomol.

[CR42] Xiao C, Hu J, Ao YT, Cheng MX, Gao GJ, Zhang QL, He GC, He YQ (2016). Development and evaluation of near-isogenic lines for brown planthopper resistance in rice cv. 9311. Sci Rep.

[CR43] Yamasaki M, Yoshimura A, Yasui H (2003). Genetic basis of ovicidal response to whitebacked planthopper (*Sogatella furcifera* Horváth) in rice (*Oryza sativa* L.). Mol Breeding.

[CR44] Yoshida S, Forno DA, Cock JH (1971). Laboratory manual for physiological studies of rice.

[CR45] Zhang Y, Gang Q, Qianqian M, Minyi W, Xinghai Y, Zengfeng M, Haifu L, Chi L, Zhenjing L, Fang L (2020). Identification of major locus *Bph35* resistance to brown planthopper in rice. Rice Sci.

[CR46] Zhao Y, Huang J, Wang Z, Jing S, Wang Y, Ouyang Y, Cai B, Xin XF, Liu X, Zhang C, Pan Y, Ma R, Li Q, Jiang W, Zeng Y, Shangguan X, Wang H, BoDu, Zhu L, Xu X, Feng Y-Q, He SY, Chen R, Zhang Q, He G (2016). Allelic diversity in an NLR gene *BPH9* enables rice to combat planthopper variation. Proc Natl Acad Sci USA.

